# Urban malaria vector bionomics and human sleeping behavior in three cities in Senegal

**DOI:** 10.1186/s13071-023-05932-9

**Published:** 2023-09-19

**Authors:** Abdoulaye Diop, Fatou Ndiaye, Katherine Sturm-Ramirez, Lassana Konate, Massila Senghor, El Hadji Diouf, Abdoulaye Kane Dia, Seynabou Diedhiou, Badara Samb, Doudou Sene, Sarah Zohdy, Ellen Dotson, Mame Birame Diouf, Valerie Koscelnik, Lilia Gerberg, Abdoulaye Bangoura, Ousmane Faye, Tiffany Clark, El Hadji Amadou Niang, Joseph Chabi

**Affiliations:** 1 U.S. President’s Malaria Initiative (PMI) Abt Associates/VectorLink Project, Dakar, Senegal; 2 Laboratoire d’Ecologie Vectorielle et Parasitaire, Faculté Des Sciences et Techniques, Université Cheikh Anta Diop, Dakar, Sénégal; 3 U.S. President’s Malaria Initiative, Malaria Branch, Centers for Disease Control and Prevention (CDC), Atlanta, GA USA; 4 National Malaria Control Programme, Dakar, Senegal; 5 U.S. President’s Malaria Initiative, United States Agency for International Development (USAID), Dakar, Senegal; 6 U.S. President’s Malaria Initiative, United States Agency for International Development (USAID), Washington, DC USA; 7 U.S. President’s Malaria Initiative, Abt Associates/VectorLink Project Rockville, Rockville, DC USA

**Keywords:** Urban malaria, Outdoor sleeping, Daaras, Malaria transmission, Insecticide-treated net (ITN), Malaria insights

## Abstract

**Background:**

Malaria is endemic in Senegal, with seasonal transmission, and the entire population is at risk. In recent years, high malaria incidence has been reported in urban and peri-urban areas of Senegal. An urban landscape analysis was conducted in three cities to identify the malaria transmission indicators and human behavior that may be driving the increasing malaria incidence occurring in urban environments. Specifically, mosquito vector bionomics and human sleeping behaviors including outdoor sleeping habits were assessed to guide the optimal deployment of targeted vector control interventions.

**Methods:**

Longitudinal entomological monitoring using human landing catches and pyrethrum spray catches was conducted from May to December 2019 in Diourbel, Kaolack, and Touba, the most populous cities in Senegal after the capital Dakar. Additionally, a household survey was conducted in randomly selected houses and residential Koranic schools in the same cities to assess house structures, sleeping spaces, sleeping behavior, and population knowledge about malaria and vector control measures.

**Results:**

Of the 8240 *Anopheles* mosquitoes collected from all the surveyed sites, 99.4% (8,191) were *An. gambiae* s.l., and predominantly *An. arabiensis* (99%). A higher number of *An. gambiae* s.l. were collected in Kaolack (77.7%, *n* = 6496) than in Diourbel and Touba. The overall mean human biting rate was 14.2 bites per person per night (b/p/n) and was higher outdoors (15.9 b/p/n) than indoors (12.5 b/p/n). The overall mean entomological inoculation rates ranged from 3.7 infectious bites per person per year (ib/p/y) in Diourbel to 40.2 ib/p/y in Kaolack. Low anthropophilic rates were recorded at all sites (average 35.7%). Of the 1202 households surveyed, about 24.3% of household members slept outdoors, except during the short rainy season between July and October, despite understanding how malaria is transmitted and the vector control measures used to prevent it.

**Conclusion:**

*Anopheles arabiensis* was the primary malaria vector in the three surveyed cities. The species showed an outdoor biting tendency, which represents a risk for the large proportion of the population sleeping outdoors. As all current vector control measures implemented in the country target endophilic vectors, these data highlight potential gaps in population protection and call for complementary tools and approaches targeting outdoor biting malaria vectors.

**Graphical Abstract:**

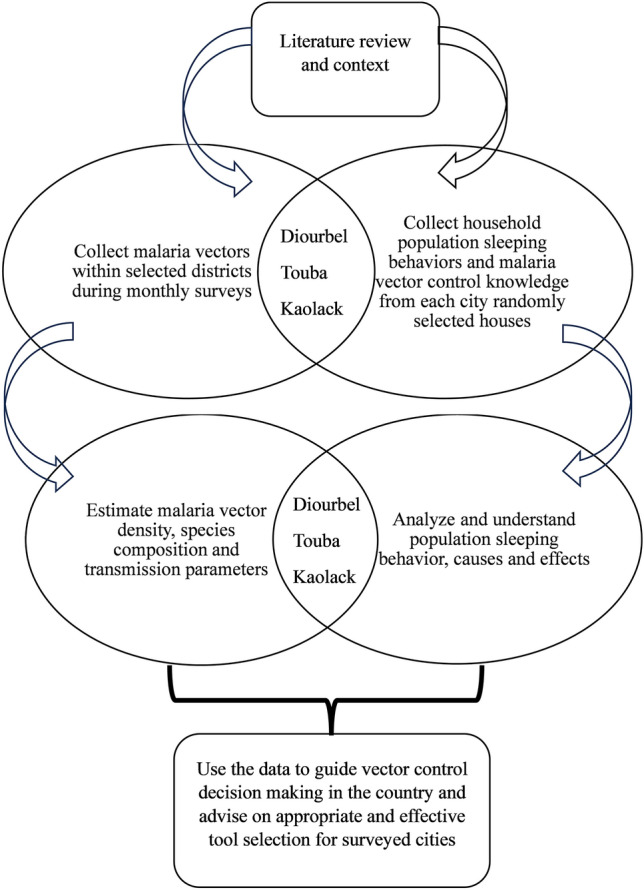

**Supplementary Information:**

The online version contains supplementary material available at 10.1186/s13071-023-05932-9.

## Background

In Senegal, malaria remains endemic and represents a major cause of morbidity and mortality, making malaria control a high priority for the government. Over the past two decades, the government of Senegal, supported by its partners and key stakeholders, has worked to reduce malaria burden in affected populations. Proven effective interventions including malaria vector control and case management have been implemented within the country, enabling a substantial decrease in malaria cases between 2008 and 2019 [1–3]. The country remains a leader in piloting and scaling up new recommendations and innovative strategies. In 2016, the National Malaria Control Programme (NMCP) adopted a National Strategic Plan (NSP) which aimed to achieve malaria pre-elimination in low transmission zones by 2020. To achieve this goal, different interventions have been implemented across the country. Mass campaigns and routine distribution of insecticide-treated nets (ITNs) are implemented countrywide to protect the population, including children under 5 years of age and pregnant women, who are at greatest risk. The country distributed 9.3 million standard ITNs during the 2019 mass distribution campaign, representing coverage of 95% of targeted households. During the 2022 mass ITN distribution campaign, 14,242,000 ITNs, including both standard and new types of ITNs, were distributed across the country, for mean coverage of approximately 98%. Additionally, indoor residual spraying (IRS) has been implemented in selected health districts since 2007 either by the government of Senegal or by partners such as the U.S. President’s Malaria Initiative (PMI). Standard case management interventions are implemented nationwide, with case investigation and reactive case detection undertaken in low transmission areas. The regions of highest transmission (Tambacounda, Kolda, and Kedougou) receive seasonal malaria chemoprevention (SMC) and are prioritized for proactive community-level malaria case management. As a result of these interventions, parasitemia in children under 5 years of age has fallen from 6% nationwide in 2008 to less than 1% nationwide in 2016, demonstrating a decline in transmission [1].

Malaria transmission is concentrated during the rainy season between July and November, with a peak in October and November at the end of the rainy season, where stagnant water facilitates vector population increases. Malaria incidence is stratified, with moderate to high malaria incidence in the southeast and decreased incidence toward the northern part of the country [4]. *Anopheles gambiae* sensu lato is the primary malaria vector across the country, and *Anopheles funestus* s.l. is a secondary vector occurring in specific sites which have been characterized in recent years [5–8]. However, despite all the intervention efforts and gains made, malaria remains a public health problem in certain areas of the country, including urban areas where seasonal transmission is affected by rainfall and persistent flooding. Furthermore, increased economic development due to overcrowding and urban expansion and subsequent environmental changes such as lack of water drainage systems and limited use of malaria preventive measures have led to an increase in malaria in urban areas. This is of concern across the African continent due to growing urbanization trends in all countries, including Senegal, invasion of new vectors such as *Anopheles stephensi*, and the increased risk of uncontrolled malaria cases [9–15]. Therefore, the characterization of transmission dynamics in urban areas is crucial to maintaining progress in Senegal toward targeted and sustainable control.

Thus, to support the NMCP to better select and implement appropriate vector control strategies targeting urban settings, a landscape analysis was conducted from May 2019 to January 2020 to identify entomological and human behavioral risk factors in Diourbel, Kaolack, and Touba, the three most populous cities after Dakar, and high contributors to malaria cases observed in Senegal.

## Methods

### Study design

**Fig. 1:**
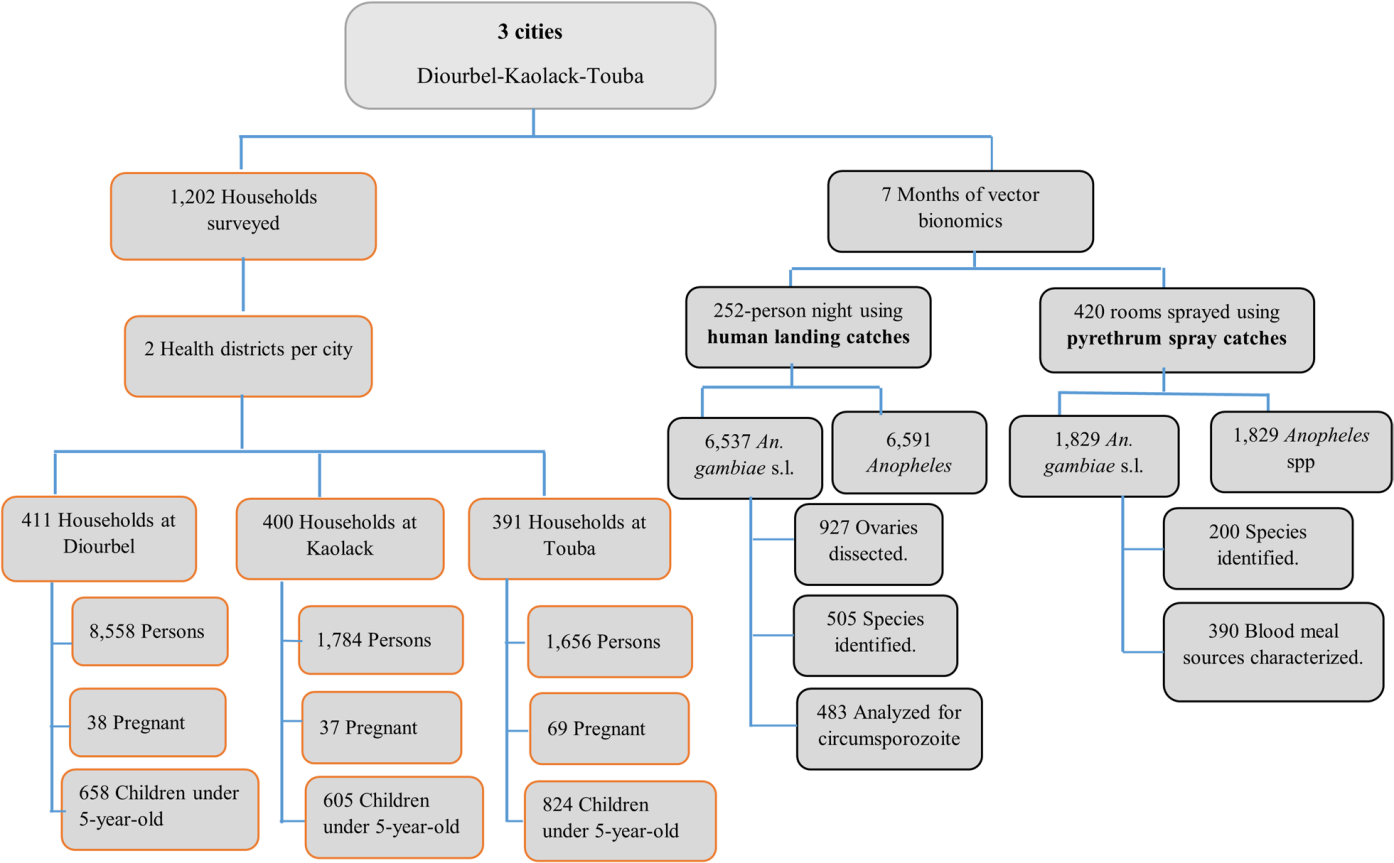
Workflow demonstrating study design and sample sizes across all three cities

The overall study design and outcome are summarized in Fig. 1, presenting the different approaches used to address the study objective: adult vector surveillance, human population behavioral questionnaire, and malaria prevention knowledge. In this study, the adult vector and human behaviors are described, as these two factors are related. Monthly adult entomological data were collected to determine vector density, biting times, feeding preferences, species composition, and infection rate. Human population behavior was recorded through a household questionnaire to estimate the attitudes of inhabitants toward malaria and vector control measures implemented by the country (Fig. 1).

### Study sites

**Fig. 2:**
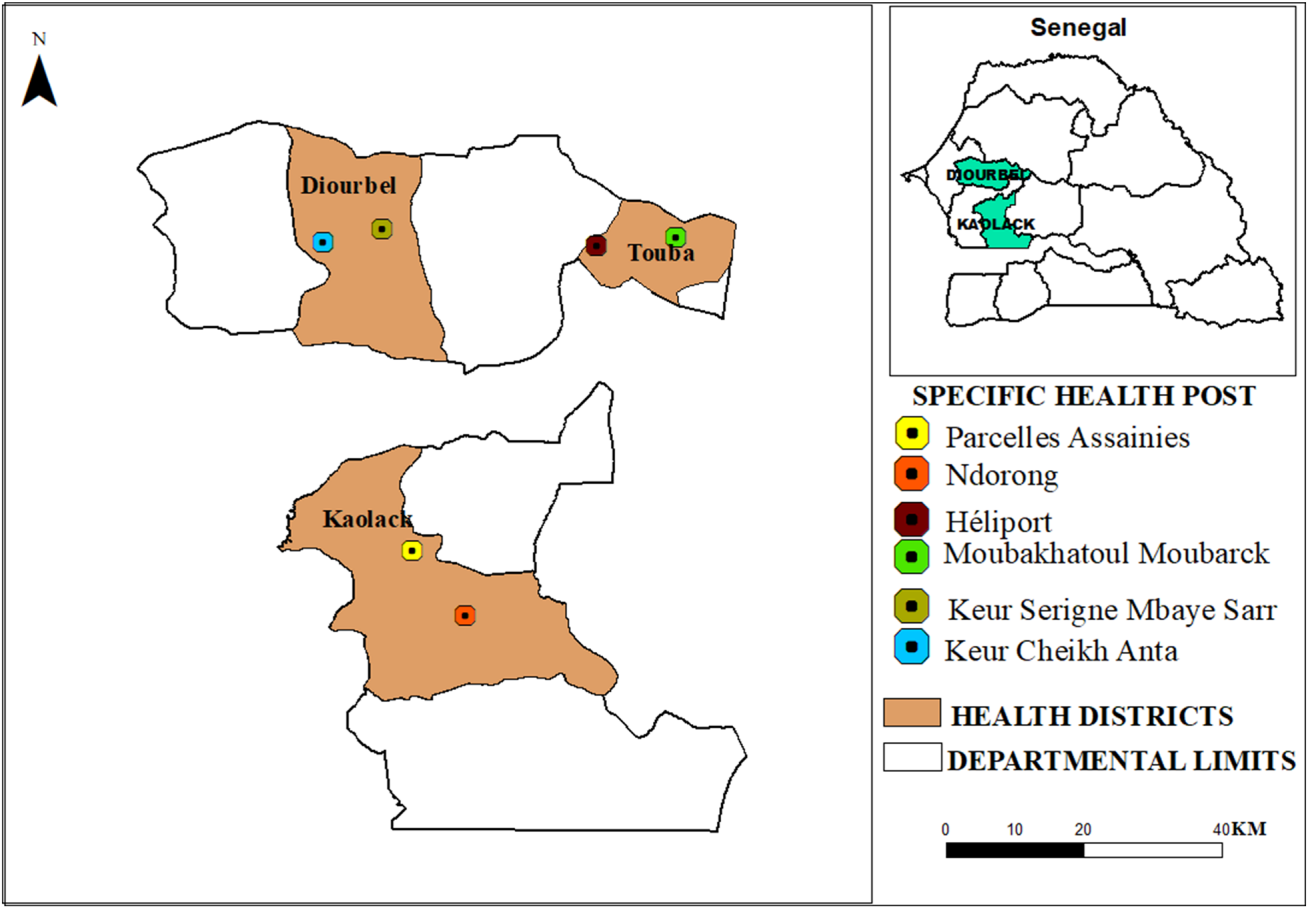
Geographical location of the cities investigated for both entomological monitoring and household surveys

The study was conducted from May to December 2019 in Diourbel, Touba, and Kaolack. All three cities are in the western part of the country and in the Sudano-Sahelian geographical zone, where malaria remains hyperendemic (Fig. 2). The city of Diourbel has the highest population density, with a mean of 20.8 inhabitants per house, compared to 4.5 in Touba and 4.2 in Kaolack. However, vulnerable populations such as children under 5 years of age and pregnant women were higher in Touba and Kaolack than in Diourbel. For each city, the sites monitored were selected from health posts within the main city or in the surroundings (Fig. 1).

Diourbel (14° 39′ 18″ N, 16° 13′ 53″ W) is about 145 km from Dakar, with an estimated population of 322,762 inhabitants according to the most recent census (2019) conducted after the latest ITN mass distribution campaign [2]. With a mean temperature above 40 °C during the long dry season (from November to June) and annual average rainfall of 486 mm between July and October, Diourbel is one of the central peanut-growing areas, where traditional peanut oil production and beverage and perfume production are the main economic activities. Moreover, the fossil valley crossing the town has higher rainfall and allows urban crop cultivation throughout the year, including vegetables such as cabbages and mint flower, but also creates a suitable habitat for *Anopheles* vectors and the persistence of malaria, with recorded incidence of 10.3, 5.6, and 4.0 cases per 1000 inhabitants in 2018, 2019, and 2020, respectively [1, 2, 16].

Touba (14° 51′ 00″ N, 15° 53′ 00″ W) is about 194 km from Dakar and represents the second most populous city of Senegal after the capital. From the most recent census, the population was estimated at 906,514 inhabitants settled in an area of 120 km^2^, with average annual rainfall of 464 mm. Although the temperature and the average rainfall are similar to those observed in Diourbel, Touba displays higher malaria incidence than Diourbel, with 15.9, 11.8, and 14.0 cases per 1000 inhabitants in 2018, 2019, and 2020, respectively [1, 2, 16]. Touba is a major Senegalese religious center where many annual events with high population movement occur, especially during the Grand Magal, when approximately four million pilgrims from all around the country and abroad converge in Touba. As the religious capital of the Mouride brotherhood, Touba is also characterized by a significant presence of traditional residential Koranic schools called Daaras, where pupils aged 5–15 years live and study.

The third city is Kaolack (14° 10′ 00″ N, 16° 05′ 00″ W), located 192 km from Dakar, which had an estimated population of 380,010 inhabitants during the most recent census. Kaolack is the heart of the peanut-growing basin and is located at the crossroads of the trans-Gambian and National 1 roads. The main economic activities in the area include agriculture, primarily peanut cultivation and trading, which brings in populations from various localities of the country and from Gambia. The city is characterized by a poor water drainage system with open canals and several shallows and valleys, creating opportunities for mosquito breeding. The average rainfall is 776 mm, with malaria incidence of 20.7, 7.7, and 8.7 cases per 1000 inhabitants in 2018, 2019, and 2020, respectively [1, 2, 16].

### Vector bionomics monitoring

Adult mosquitoes were collected using human landing catches (HLCs), conducted by paid collectors, indoors and outdoors in six selected houses per city. Collection was conducted hourly from 8:00 p.m. to 6:00 a.m. on two consecutive nights per month in May and from July through December 2019 in Diourbel, and from July through December 2019 in Kaolack and Touba. Subsamples (about 30% overall) of morphologically identified *An. gambiae* s.l. were randomly selected per collection hour per month and ovary-dissected for parity. Additionally, indoor resting mosquitoes were collected using pyrethrum spray catches (PSC) between 6:00 a.m. and 8:00 a.m. in 20 randomly selected rooms for each surveyed city each month. All mosquitoes collected were morphologically identified to the genus level. Then *Anopheles* mosquitoes were subsequently identified to the species or species complex/group level using classical morphological identification keys [17, 18]. All the *Anopheles* females collected by PSC were sorted by species and by abdominal status as unfed, blood-fed, half-gravid, or gravid. *Anopheles* females from both collection methods were stored individually in numbered tubes containing silica gel for further laboratory processing.

### Laboratory processing

Subsamples of 729 *An. gambiae* s.l. collected by HLC and PSC were analyzed to determine the members of the *An. gambiae* s.l. complex. The genomic DNA of individual mosquitoes was extracted as described by Collins et al. [19], and the members of the *An. gambiae* species complex were identified using the short interspersed nuclear element-based polymerase chain reaction (SINE PCR) protocol described by Santolamazza et al. [20].

Subsamples of 483 *An. gambiae* s.l. females collected by HLC from all surveyed cities were individually screened by circumsporozoite enzyme-linked immunoassay (ELISA) to detect the presence of the *Plasmodium falciparum* sporozoites as described by Wirtz et al. [21]. The blood meal sources of the blood-fed females of *An. gambiae* s.l. collected by PSCs were determined using the procedure described by Beier et al. [22]. The anthropophilic rate was calculated as the ratio of females that fed on humans to the total number of blood sources identified. Mixed blood meals were counted for each of the hosts involved, two and three times for double and triple meals, respectively.

### Household survey

#### Preparation of the survey and conception of tools

A steering committee including key malaria stakeholders in the country and partners was set up to guide the household assessment. The protocol for the study was shared, reviewed, and approved prior to the training on the appropriate data collection procedures for the field investigators. A data collection tool was developed and integrated into tablets using the Open Data Kit (ODK) system. Household structures and other environmental factors, sleeping behaviors, and existing household vector control practices were the main characteristics considered in the questionnaire developed for the survey (Table 1).

**Table 1:** Summary of indicators for household survey

Data category	Expected outcomes
Household demographics	Number of household members Number of children < 5 Number of pregnant women
Structure	Number of eligible structures Type of roof and wall building materials Number of windows and doors Presence of curtains
Sleeping spaces	Location (inside and/or outside) Number/type of beds (mats, other types of supports)
Sleeping behavior	Outdoor sleeping frequency/times Number of people per sleeping space Reasons for sleeping outside
Vector control practices	Type of vector control practices Number/type of hanging nets Number of people sleeping under a net (pregnant women/children < 5)

#### Field data collection

Five investigators were recruited in each city and trained for data collection using a questionnaire-based digital form and tablets and supervised by the district health director. The investigators were recruited within each community of residents and spoke each city’s local language. Two hundred households were randomly sampled using a systematic random sampling approach in the catchment area of each of the two selected health posts per city, for a total of 1200 households across the three cities. Within each household, data were collected from either the head of household or any available adult member living in the surveyed household in the absence of the head of household. Each data collector was assigned to visit 20 households per day. Specific data collection was conducted in Koranic teaching schools, called Daaras in Senegal, hosting children between the age of 5 and 15 years. The Koranic teacher was the respondent at each of the schools surveyed. At least five Daaras from each area were selected by the health nurse, and each Daara was considered a household.

A subsample of 100 households from each health post catchment area included in the survey were selected to participate in the knowledge, attitudes, and practice (KAP) survey on malaria transmission factors and vector control tools and interventions, for a total of 600 households across the three cities.

### Statistical analysis

Linear regression using SPSS Statistics 25, was used to compare mean of human biting rates and indoor resting densities over the collection period and between the different study cities. All tests were performed at the 5% significance level. Vector population percentages were calculated per city using the proportion of each species recorded. Similarly, the survey indicators were calculated using the proportion of each indicator divided by the total numbers collected. The mean human biting rate (HBR) and entomological inoculation rate (EIR) for each city were calculated as the average monthly HBRs and EIRs, respectively.

## Results

### Entomological vector bionomics monitoring

#### Species composition

Overall, a total of 8420 *Anopheles* mosquitoes were collected by HLC and PSC across all three cities. All the collected *Anopheles* specimens belonged to *An. gambiae* s.l., *An. funestus* s.l., and *An. pharoensis*.

**Fig. 3:**
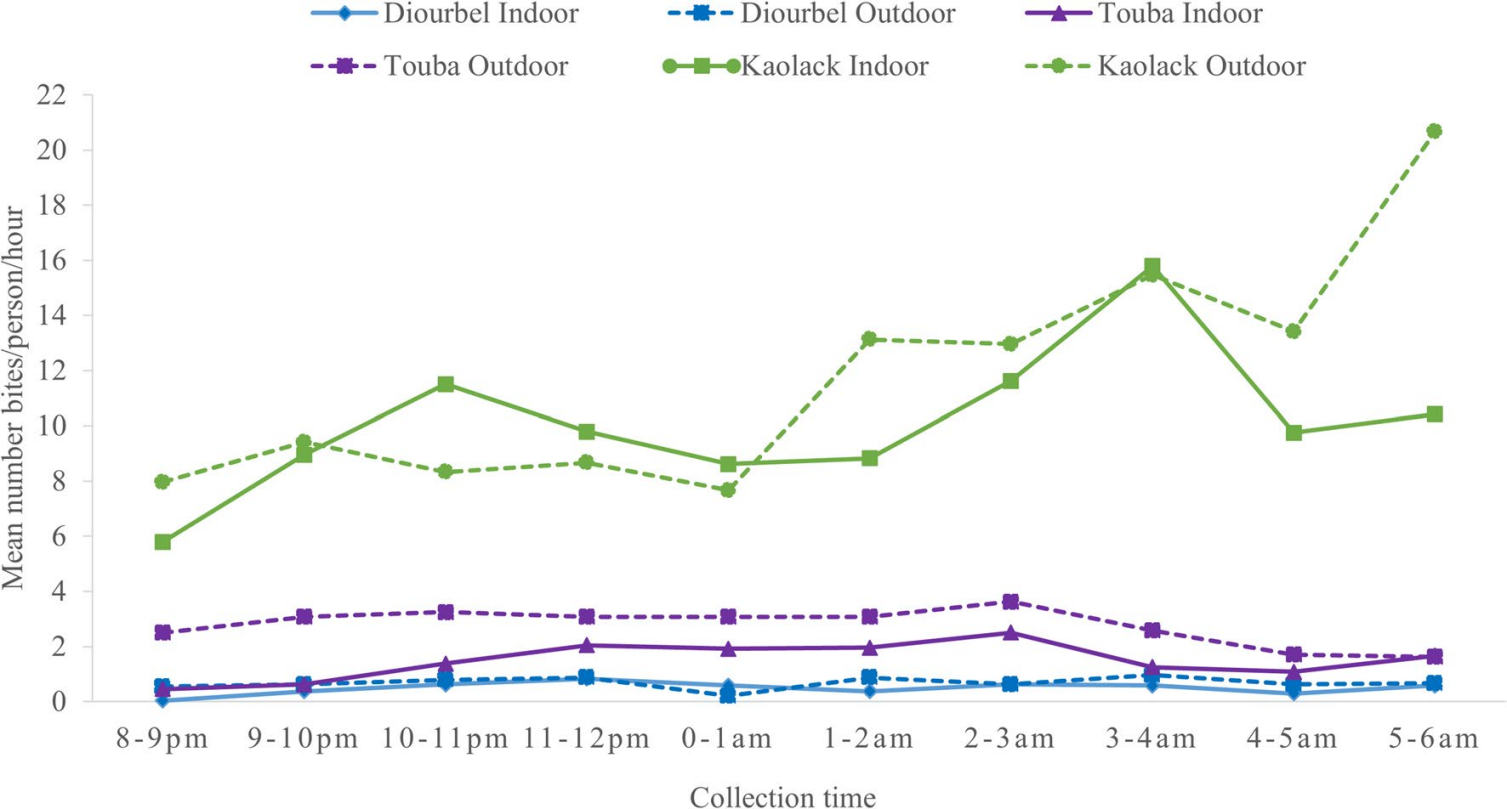
Mean indoor and outdoor hourly biting rates of *An. gambiae* s.l. in Diourbel, Touba, and Kaolack

With 77.7% (*n* = 6546) of the total collection, the city of Kaolack had the highest proportion of *Anopheles* (Figs. 2 and 3), followed by Touba and Diourbel, where 1382 (16.4%) and 492 (5.8%) specimens were collected, respectively. Overall, *An. gambiae* s.l. (*n* = 8366/8420; 99.4%) was the most common *Anopheles* collected across the three study areas. Only a single specimen of *An. funestus* s.l. was collected by HLC in Touba, and three *An. pharoensis* were caught by HLC in Touba and 50 in Kaolack. Of the total *An. gambiae* s.l. collected in the three cities, 927 were dissected, with an overall mean parity rate of 35.5%, including 31.6% (*n* = 37/117) in Diourbel, 41.1% (*n* = 140/341) in Touba, and 33.7% (158/469) in Kaolack (Table 2, Additional file 1).

**Table 2:** *Anopheles* species composition and parity rates per city

	Cities/collection methods	Total *Anopheles* collected				Parity		Total analyzed		
		*An. gambiae* s.l.	*An. pharoensis*	*An. funestus* s.l.	Total collected	Total ovary dissected	Total parous (%)	*An. arabiensis* (%)	*An. coluzzii* (%)	Total identified
HLC	Diourbel	281	0	0	281	117	37 (31.6)	276 (100)	0 (0.0)	276
	Touba	1016	3	1	1020	341	140 (41.1)	144 (99.3)	1 (0.7)	145
	Kaolack	5240	50	0	5290	469	158 (33.7)	282 (99.3)	2 (0.7)	284
PSC	Diourbel	211	0	0	211	nc	nc	nc	nc	nc
	Touba	362	0	0	362	nc	nc	nc	nc	nc
	Kaolack	1256	0	0	1256	nc	nc	nc	nc	nc
	Total per species	8366 (99.4%)	53 (0.6%)	1 (0.01%)	8420	927	335 (35.5%)	702 (99.6%)	3 (0.4%)	705

*nc* not completed

*Anopheles gambiae* s.l. was the only *Anopheles* species found resting indoors (PSC) in all three cities (Table 2).

Molecular identification of the members of the *An. gambiae* s.l. complex revealed that during the study period, the complex was represented only by *An. arabiensis* and *An. coluzzii,* with the notable absence of *An. gambiae* sensu stricto (s.s.). Of the 729 *An. gambiae* s.l. mosquito species identified, 24 failed to amplify. Overall, *An. arabiensis* was the only species of the complex found in Diourbel, and the most predominant in the two other cities (Touba and Kaolack) regardless of the collection method. *Anopheles coluzzii* was found only in Kaolack and Touba (Table 2).

#### Host-seeking behavior and biting cycle of *An. gambiae* s.l.

During the study period, *An. gambiae* s.l. showed predominantly outdoor biting behavior in all three cities, especially in Touba. About 61% of the mosquitoes were collected outdoors in Diourbel, 54% in Kaolack, and 65% in Touba. The *An. gambiae* s.l. complex presented the highest hourly biting rates both indoors and outdoors in Kaolack (Fig. 2, Additional files 1 and 2), where the biting rate was higher indoors during the first half of the night from 9:00 p.m. to 1:00 a.m. and shifted during the second half of the night, with more females biting outdoors despite a notable indoor biting peak between 3:00 a.m. and 4:00 a.m. The HBR of *An. gambiae* s.l. was lower in Kaolack than in either Diourbel or Touba (Fig. 2). In these two cities, the biting rate of *An. gambiae* s.l. females was nearly the same, with fewer than three bites per person per hour (b/p/h) both indoors and outdoors. The biting rate increased slightly between 8:00 p.m. and 12:00 a.m. in both Diourbel and Touba, then stalled through the remainder of the night.

The mean monthly HBR was significantly higher in Kaolack (R Square = 0.994; Std. Error = 5.89; df 1 = 2; df 2 = 4, *P* < 0.0001, *P* < 0.001), with 37 bites per person per night (b/p/n), than in Touba (7 b/p/n) and Diourbel (1 b/p/n) (Fig. 3, Additional files 1 and 2). Furthermore, the outdoor HBR was significantly higher in Touba (65% of the collections outdoors) (R Square = 0.981; Std. Error = 0.61; df 1 = 1; df 2= 4; *P* < 0.0001, *P* < 0.001), particularly during the month of September when the peak density was recorded, while in Diourbel and Kaolack, the trends were fairly similar indoors and outdoors. Overall, two specific biting tendencies were observed during the collection period: a single peak during the rainy season, and lower density before and after the peak, corresponding to the start and end of the dry season, respectively. The HBRs recorded during the dry season were four, 30, and 44 times lower than those of the rainy season in Diourbel, Touba, and Kaolack, respectively (Fig. 3).

#### Indoor resting density of *An. gambiae* s.l.

**Fig. 4:**
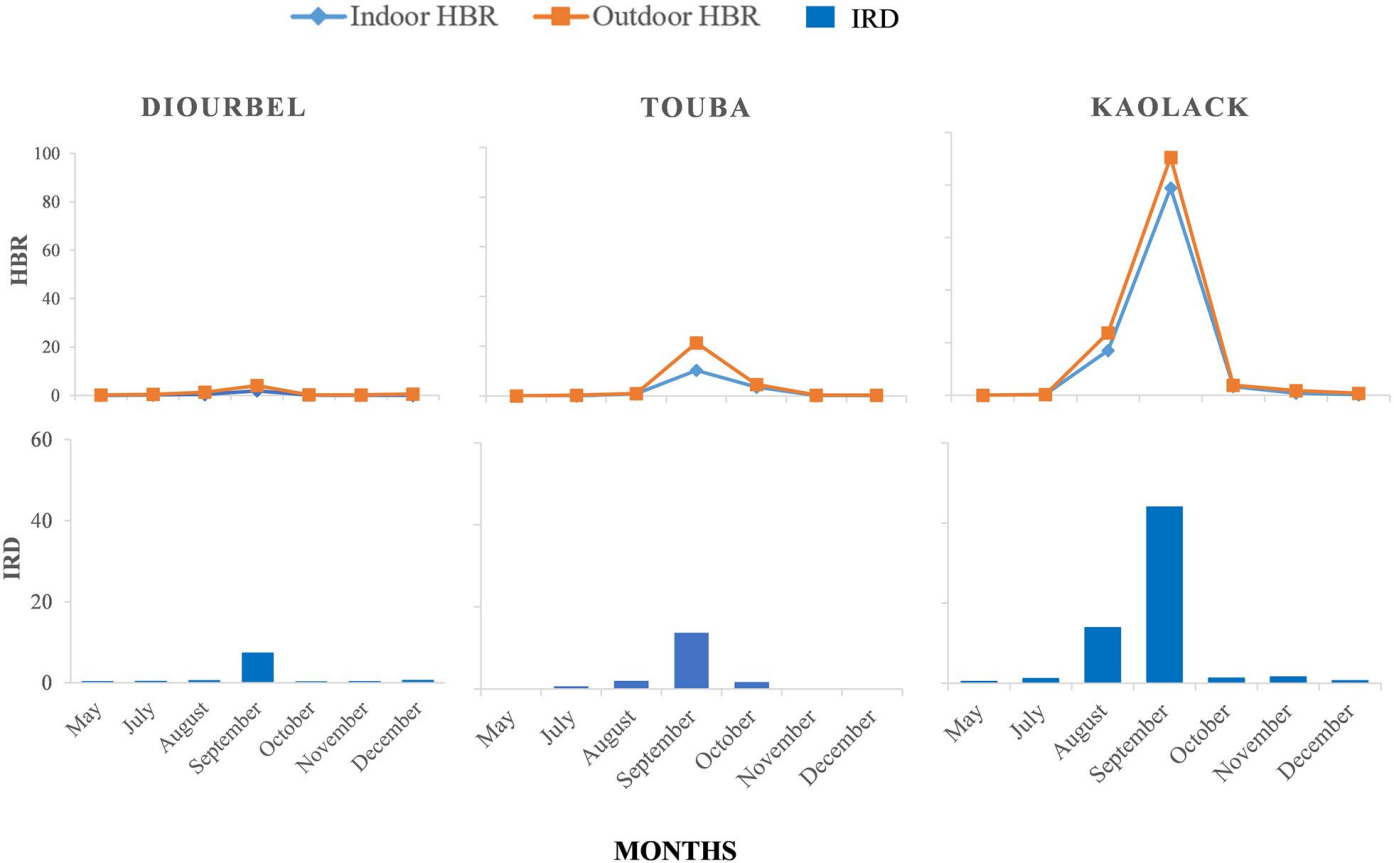
Indoor and outdoor HBRs and IRD of *An. gambiae* s.l. collected per site during the collection period

The analysis of the resting behavior of *An. gambiae* s.l. showed that the mean indoor resting density (IRD) was significantly (R Square = 0.952; Std. Error = 4.33; df 1 = 2; df 2 = 4; *P* < 0.02, *P* < 0.001) lower in Diourbel and Touba, with only one female per room, than in Kaolack, with eight females per room (Fig. 4, Additional files 1 and 2). The highest IRD was recorded during the rainy season, with a mean of 15 females per room in Kaolack. During the dry season, less than one female of *An. gambiae* s.l. per room was collected in any of the surveyed districts.

The IRD increased gradually in all districts from August, at the start of the rainy season, and peaked in September, with an average of 44 females collected per room in Kaolack. However, a drastic drop in mosquito resting density was observed as early as October, with less than two females/room in all districts.

#### *Plasmodium falciparum* infection rate and entomological inoculation rate of *An. gambiae* s.l.

Overall, the *P. falciparum* infection rates of *An. gambiae* s.l. were relatively low and not significantly different between the cities.

However, due to differences in HBR, the annual EIR of *An. gambiae* s.l. was lowest in Diourbel, with 3.65 infective bites per person per year (ib/p/y), compared to Touba at 7.31 ib/p/y, and highest in Kaolack with 40.21 ib/p/y (Table 3).

**Table 3:** Entomological inoculation rate of *An. gambiae* s.l. in Diourbel, Touba, and Kaolack

City	HBR	No. ELISA-tested	No. CSP-positive	SR	EIR (b/p/n)	Annual EIR (b/p/y)
Diourbel	1.301	203	1	0.0049	0.01	3.65
Touba	1.792	104	1	0.0096	0.02	7.31
Kaolack	10.058	176	2	0.0114	0.11	40.21

*CSP* circumsporozoite, *SR* sporozoite rate, *b/p/n* bites per person per night, *b/p/y* bites per person per year

### Household survey

A total of 1202 households were surveyed across the three cities, with 11,998 inhabitants recorded including 2117 children under 5 years and 138 pregnant women (Table 4). The mean number of household members was around 10 per house in all cities. The number of children under 5 years and pregnant women per household was similar across all districts as well (Table 4). Most of the houses surveyed were made of cement (92.5%) with a zinc roof (66.9%) and straw (6.0%). It was also noted that most houses in all three cities had windows (88.8%), with 21.2% of them protected with mesh as a mosquito barrier.

**Table 4:** Household inhabitants and ITN owners

City	No. of households surveyed	No. of inhabitants	Overall ITN owners	No. of children < 5	No. of children with ITN (%)	No. of pregnant women	No. of pregnant women with ITN (%)
Diourbel	411	4169	3575 (85.8%)	688	600 (87.2%)	38	34 (89.5%)
Touba	391	3747	744 (19.9%)	824	750 (91.0%)	63	59 (93.7%)
Kaolack	400	4082	3721 (91.2%)	605	519 (85.8%)	37	27 (73.0%)
Total	1202	11,998	8040 (67.0%)	2117	1869 (88.3%)	138	120 (87.0%)

Of the 624 individuals who participated in the household survey (215 in Diourbel, 201 in Touba, and 208 in Kaolack) and were questioned about malaria knowledge, causes, and prevention methods, there were no refusals, and nearly all (98%) knew of malaria while 64.7% considered mosquitoes as the main cause of malaria. Additionally, an ITN was the most frequently cited prevention method in all cities, followed by use of sprays and repellents. Other prevention methods proposed by the respondents included avoiding the sun, early sleeping time, use of fans, and limiting the entrance of mosquitoes into the house using by screening windows and doors.

Over 50% of sleeping spaces were shared by two people in all cities while 30% of Diourbel and Kaolack households reported sleeping spaces shared by three people. The percentage of more than five people in the same bed was as low as 2%. All sleeping spaces were checked for the presence of ITNs both indoors and outdoors, showing indoor ITN coverage of 84–92% and outdoor coverage of 83–94% across all cities. As the households possess a sufficient supply of ITNs following several mass distributions, both indoor and outdoor sleeping spaces were covered wherever the sleepers were. The percentage of household members who had slept with an ITN during the past week was high in Kaolack at 91.2% and Diourbel at 85.8%, but much lower in Touba at 19.9%. Children under the age of 5 (88.3%) and pregnant women (87.0%) had relatively high ITN utilization in all three cities. Although overall household utilization was highest in Kaolack at 91.2%, ITN utilization by pregnant women in Kaolack was the lowest, at 73.0% (Additional file 3).

Data were only collected on respondent behaviors related to time spent sleeping outside, not all household members. Of the respondents who answered the question about the sleeping location and time, 24.3% slept outside, and most of the time spent outside was in the first half of the night (before midnight). Among the three districts, Touba had the largest proportion of household members who reported having slept outside during the past week (35.6%), and of these, about 38.0% used ITNs to protect themselves while sleeping outdoors. The most common reason for sleeping outdoors was the heat inside the rooms during the long period of the dry season. However, the limited number of sleeping spaces or rooms for family members also contributed to outdoor sleeping. The other reasons mentioned included the fact that some respondents worked as security guards, while some assumed that there were more mosquito bites indoors than outdoors (Table 5, Additional file 3).

**Table 5:** Outdoor sleeping and reasons

City	Outdoor sleeping		ITNs used outdoors		Reason for sleeping outdoor				
	No. of household members	No. sleeping outside (%)	Outdoor sleeping space	No. with ITNs (%)	No. of respondents	Heat, no. (%)	Limited sleeping space, no. (%)	Limited rooms, no. (%)	Other, no. (%)
Diourbel	4169	967 (23.2%)	361	111 (30.7%)	226	126 (55.8%)	6 (2.7%)	9 (4.0%)	85 (37.6%)
Touba	3747	1334 (35.6%)	392	168 (42.9%)	201	163 (81.1%)	2 (1.0%)	4 (2.0%)	32 (15.9%)
Kaolack	4082	620 (15.2%)	422	167 (39.6%)	132	76 (57.6%)	3 (2.3%)	3 (2.3%)	50 (37.9%)
Total	11,998	2921 (24.3%)	1175	446 (38.0%)	559	364 (65.1%)	11 (2.0%)	16 (3.2%)	167 (29.9%)

#### Behavior in city Daaras

Thirty-four Daaras were visited across the three cities including nine in Diourbel, 15 in Touba, and 10 in Kaolack. A higher proportion of Daara residents slept outside in Touba (68.3%) and Kaolack (68.2%) than in Diourbel (58.0%) (Table 6, Additional file 3), and most of the outdoor sleeping occurred during the first half of the night (between 7:00 pm and 12:00 pm) in all cities (80.0%). The children often slept on a single floor mat when available or slept directly on the floor in some of the Daaras, making it difficult to provide protection using individual ITNs.

**Table 6:** Percentage of sleeping locations in Daaras

	No. Daaras visited	No. structures identified	No. rooms identified	No. people sleeping inside (%)	No. people sleeping outside (%)	Total sleepers recorded
Diourbel	9	28	33	47 (42.0)	65 (58.0)	112
Touba	15	27	66	102 (31.7)	220 (68.3)	322
Kaolack	10	21	40	77 (31.8)	165 (68.2)	242
Total	34	76	139	226 (31.4)	450 (66.6)	676

Across all cities, Daara residents slept outdoors mainly due to heat and the high number of school children. However, about 87.8% of the Daara residents slept under ITNs in Touba, 25.9% in Diourbel, and 64.5% in Kaolack.

## Discussion

A major public health concern in Senegal and globally is whether urbanization might be shifting malaria from a rural to an urban disease [13, 23]. Urban malaria has been the focus of several studies given that the implementation of rural targeted vector control tools such as IRS, larval source management (LSM), and even ITNs could be logistically challenging in cities where acceptance and use are often low due to housing types and conditions [10, 13, 24]. To better understand and prevent urban malaria, this study was designed to identify human behavior and malaria vector population dynamics which may contribute to the observed increase in malaria cases in three of the most populous cities of Senegal.

The entomological monitoring data revealed a predominance of *An. arabiensis* in all three surveyed cities, where it displayed variable seasonal density and biting behaviors. The vector is known to be present mostly in arid areas like the three cities surveyed and as already reported in the country [25, 26]. Unsurprisingly, the highest vector population density was recorded during the rainy season in all three cities, with Kaolack yielding the highest HBR over the collection period. The results generally showed that the vectors bite indoors and outdoors, with a higher outdoor tendency throughout the study period in all cities, except in Kaolack during the dry season.

The study also revealed that the vector population longevity estimated through ovary dissection and parity rates of *An. gambiae* s.l. was relatively low in all three cities and over the whole study period, suggesting young vector populations, which has critical epidemiological importance for malaria transmission risk. This could be explained by the proliferation and high productivity of suitable larval habitats, especially during the rainy season, and the high frequency and persistence of man-made water storage systems in the three cities [27]. Thus, the monitoring of vector population dynamics and larval habitats during this particular period of the year could be used to implement targeted vector control approaches, such as LSM, to supplement core interventions in urban settings wherever larval habitats are few, findable, and fixed, as recommended by the World Health Organization (WHO) [28].

*Anopheles arabiensis* was the primary vector across the study area and potentially plays the main role in malaria transmission in the surveyed cities. The low anthropophagy and longevity of *An. arabiensis* populations may explain the level of malaria transmission across the study area, with low *P. falciparum* (*Pf*) sporozoite infections recorded, although the small number of mosquitoes analyzed may limit the probability of detecting *Pf*-positive specimens. The annual EIRs recorded in all cities ranged from 3.7 ib/p/y in Diourbel to 40.2 ib/p/y in Kaolack, which may be considered low in comparison to rural and even urban settings elsewhere [9, 29]. Though the transmission could be low due to the low physiological age of host-seeking females, these data could contribute to appropriate measures for malaria elimination in these cities using closed monitoring and targeted vector control tools in putative hotspots. Furthermore, the incidence of urban malaria is likely underestimated due to the low level of reporting among private-sector health service providers and to the assumption that several control and prevention tools are easily accessible to urban populations [10, 30]. The higher concentration of both public and private health facilities in urban settings creates ideal conditions for urban populations to seek care and treat themselves against several vector-borne diseases including malaria. Therefore, additional considerations are needed to distinguish autochthonous cases from those imported from surrounding rural and peri-urban areas, and proactive and reactive vector control measures for urban populations, which likely display the lowest immunity [31].

The investigation of human sleeping behaviors along with vector dynamics revealed that during the dry season, members of most of the surveyed households spent more time outdoors during the night due to the heat, exposing themselves to vector bites and sleeping in conditions that are not ideal for ITN use. The crowding of youth was seen in several Daaras in Touba, where pupils spent a good deal of time outdoors, especially during nighttime learning the Koran, generally from 6:00 p.m. to 9:00 p.m. and sometimes late in the night, or early in the morning (5:00 a.m. to 7:00 a.m.), times when *An. gambiae* s.l. was actively biting. The time spent outdoors and outdoor sleeping behaviors of a large number of inhabitants, unprotected by any vector control tool, could increase the risk of malaria. This highlights the need for targeted complementary control interventions specific to Daaras, which are quite different living quarters from traditional houses. Recent studies have reported the importance of simultaneous human and vector behavior assessments as an approach to better understand the bidirectional feedback between human and vector behaviors and how they may shift in response to the deployment of vector control tools such as IRS and ITNs [32–34].

In this study, knowledge regarding the sources of malaria transmission and preventive measures was high in all three urban areas, which could be an important asset to support community-based vector control strategies. Even though other factors such as housing characteristics, population behavior, or agricultural activities could impact vector density and species composition and/or behavior, knowledge of disease transmission and prevention could support the implementation and impact of vector control strategies [35]. In addition, as reported in the country’s Demographic and Health Survey (DHS) and Malaria Indicator Survey (MIS) 2020–2021, overall household ITN coverage and usage were relatively low across the country, with a mean of 58% and 46%, respectively. In particular, these were lower in urban (51% coverage and 38% usage) than rural areas (63% coverage and 53% usage) [36]. Furthermore, ITN use was higher in Diourbel and Kaolack than in Touba, where only 19.9% of the distributed ITNs were utilized, which is a concern for the NMCP and its partners in their attempt to eliminate the disease. Though Islam is the main religion of the country (92–95% of the population), the city of Touba has the particularity of being considered a holy city in Senegal, hosting many Koranic schools where the sleeping conditions of the pupils may not be appropriate for ITN use. However, children under 5 years and pregnant women were still well protected with ITNs in the households of each city. Near-optimal ITN utilization (defined as ≥ 80% use) by pregnant women and children under 5 was documented, except in Kaolack, where ITN utilization by pregnant women was 73%.

Touba had the highest number of Daaras, though only selected Daaras were investigated. About 59% of the inhabitants in the Daaras across the three cities slept outdoors, particularly during the first half of the night. The ITN coverage and use in Daaras warrants further investigation, as ITNs are usually designed for individual beds and are not appropriate for Daaras due to the number of children, group sleeping behavior, and limited sleeping spaces. Furthermore, additional interventions need to be identified to address the vector control gap caused by outdoor sleeping and learning behaviors during the peak biting hours by vectors. These complementary interventions should align with the WHO recommendations and country target of achieving at least 80% ITN coverage and use for all at-risk populations to reduce vector populations. High ITN coverage and use may also contribute to a significant reduction in malaria cases and protect the entire population, including non-ITN users [37, 38]. Among the three cities, Diourbel showed the highest ITN use, with about 80% of the population found sleeping under ITNs. This could contribute to the low transmission observed in Diourbel among the three cities surveyed. Furthermore, additional education about the uptake and use of ITNs during the transmission period could be implemented within the urban population to reduce the contact between humans and vectors. However, depending on the level of transmission occurring in urban areas relative to other settings, prevention and control strategies may need to be modified extensively to adapt to the heterogeneity in environments and human and vector dynamics [23].

Although the data recorded in this study would support the country regarding vector control strategies, a few limitations can be noted. For example, no data were collected on human or vector behavior associated with ITN use to enable quantification of the true gaps in protection provided by vector control interventions, and to identify targeted approaches to ensure the effectiveness of control tools on malaria transmission. An additional limitation was that, although household selection was randomized, the heterogeneity in population and housing density across the cities could affect the generalizability of these findings.

## Conclusion

Overall, given the complexities of the three cities surveyed and the variation in human and vector dynamics observed, a targeted, cost-effective, and sustainable community-based vector control intervention can be developed for each specific context based on the human and vector behavior data provided here, and integrated with available epidemiological data to better characterize malaria hotspots and risk across the study areas. To better guide the NMCP’s decision-making, the results obtained from this study could help to fine-tune potential intervention combination scenarios to be undertaken, including (i) personal protection measures for Daaras using topical repellents for schoolchildren during learning hours, (ii) house improvement strategies to make structures and sleeping spaces mosquito-proof by screening doors and windows and/or impregnating curtains for doors and windows to reduce mosquito entry and longevity, (iii) use of treated eave tubes, (iv) tailoring of available ITNs to adapt to sleeping spaces for children in Daaras, and (v) implementation of LSM in Touba and in Diourbel as a complementary vector control tool to ITNs to target outdoor biting *An. arabiensis* vectors. However, future larval surveillance and habitat mapping will be needed to provide information on spatial heterogeneity in vector distribution data to determine the feasibility of LSM.

### Supplementary Information

**Additional file 1. **Hourly biting rates and parity rates.

**Additional file 2. **Human biting rates and indoor resting density per city.

**Additional file 3. **Household survey results.

## Data Availability

All data generated or analyzed during this study are included in this published article and its supplementary information files online.

## Abbreviations

EIR: Entomological inoculation rate
HLC: Human landing catch
PSC: Pyrethrum spray catch
IRD: Indoor resting density
IRS: Indoor residual spraying
ITN: Insecticide-treated net
NMCP: National Malaria Control Programme
PCR: Polymerase chain reaction
SINE: Short interspersed nuclear element
WHO: World Health Organization

## References

[1] NMCP (2018). Bulletin epidemiologique annuel du paludisme au Senegal.

[2] NMCP (2019). Bulletin epidemiologique du paludisme au Senegal.

[3] WHO (2021). World malaria report.

[4] Seck MC, Thwing J, Fall FB, Gomis JF, Deme A, Ndiaye YD (2017). Malaria prevalence, prevention and treatment seeking practices among nomadic pastoralists in northern Senegal. Malaria J.

[5] PMI/VL (2017). US President’s Malaria Initiative, VectorLink Senegal Project Entomology Annual Report.

[6] PMI/VL (2018). US President's Malaria Initiative, VectorLink Senegal Project Entomology Annual Report.

[7] PMI/VL (2019). US President's Malaria Initiative, VectorLink Senegal Project Entomology Annual Report.

[8] PMI/VL (2020). US President's Malaria Initiative, VectorLink Senegal Project Entomology Annual Report.

[9] Hay SI, Guerra CA, Tatem AJ, Atkinson PM, Snow RW (2005). Urbanization, malaria transmission and disease burden in Africa. Nat Rev Microbiol.

[10] Keiser J, Utzinger J, Caldas de Castro M, Smith TA, Tanner M, Singer BH (2004). Urbanization in sub-Saharan Africa and implication for malaria control. Am J Trop Med Hyg.

[11] Larson PS, Eisenberg JNS, Berrocal VJ, Mathanga DP, Wilson ML (2021). An urban-to-rural continuum of malaria risk: new analytic approaches characterize patterns in Malawi. Malaria J.

[12] Robert V, Macintyre K, Keating J, Trape JF, Duchemin JB, Warren M (2003). Malaria transmission in urban sub-Saharan Africa. Am J Trop Med Hyg.

[13] WHO (2022). Global framework for the response to malaria in urban areas.

[14] Sinka ME, Pironon S, Massey NC, Longbottom J, Hemingway J, Moyes CL (2020). A new malaria vector in Africa: predicting the expansion range of *Anopheles stephensi* and identifying the urban populations at risk. Proc Natl Acad Sci U S A.

[15] Mnzava A, Monroe AC, Okumu F (2022). *Anopheles stephensi* in Africa requires a more integrated response. Malaria J.

[16] NMCP (2021). Bulletin epidemiologique annuel du paludisme au Senegal.

[17] Coetzee M (2020). Key to the females of Afrotropical Anopheles mosquitoes (Diptera: Culicidae). Malaria J.

[18] Gillies MT, Coetzee M (1987). A supplement to the *Anophelinae* of Africa south of the Sahara. Pub South Afr Inst for Med Res.

[19] Collins FH, Mendez MA, Rasmussen MO, Mehaffey PC, Besansky NJ, Finnerty V (1987). A ribosomal RNA gene probe differentiates member species of the *Anopheles gambiae* complex. Am J Trop Med Hyg.

[20] Santolamazza F, Mancini E, Simard F, Qi Y, Tu Z, della Torre A (2008). Insertion polymorphisms of SINE200 retrotransposons within speciation islands of *Anopheles gambiae* molecular forms. Malar J.

[21] Wirtz RA, Zavala F, Charoenvit Y, Campbell GH, Burkot TR, Schneider I (1987). Comparative testing of monoclonal antibodies against *Plasmodium falciparum* sporozoites for ELISA development. Bull World Health Organ.

[22] Beier JC, Perkins PV, Wirtz RA, Koros J, Diggs D, Gargan TP (1988). Bloodmeal identification by direct enzyme-linked immunosorbent assay (ELISA), tested on *Anopheles* (*Diptera: Culicidae*) in Kenya. J Med Entomol.

[23] Wilson ML, Krogstad DJ, Arinaitwe E, Arevalo-Herrera M, Chery L, Ferreira MU (2015). Urban malaria: understanding its epidemiology, ecology, and transmission across seven diverse ICEMR network sites. Am J Trop Med Hyg.

[24] Donnelly MJ, McCall PJ, Lengeler C, Bates I, D'Alessandro U, Barnish G (2005). Malaria and urbanization in sub-Saharan Africa. Malar J.

[25] Doucoure S, Thiaw O, Wotodjo AN, Bouganali C, Diagne N, Parola P (2020). *Anopheles arabiensis* and *Anopheles funestus* biting patterns in Dielmo, an area of low level exposure to malaria vectors. Malaria J.

[26] Faye O, Konate L, Mouchet J, Fontenille D, Sy N, Hebrard G (1997). Indoor resting by outdoor biting females of *Anopheles gambiae* complex (Diptera: Culicidae) in the Sahel of northern Senegal. J Med Entomol.

[27] Ndiaye F, Diop A, Chabi J, Sturm-Ramirez K, Senghor M, Diouf EH, et al. Distribution and dynamics of *An. arabiensis* larval habitats in three cities with urban malaria incidences—Diourbel, Touba and Kaolack (Senegal). Unpublished data; 2023.

[28] WHO (2013). Larval Source Management: a supplementary measure for malaria vector control: an operational manual.

[29] Saugeon C, Baldet T, Akogbeto M, Henry MC (2009). Will climate and demography have a major impact on malaria in sub-Saharan Africa in the next 20 years?. Med Trop (Mars).

[30] Byrne N (2007). Urban malaria risk in sub-Saharan Africa: where is the evidence?. Travel Med Infect Dis.

[31] Baragatti M, Fournet F, Henry MC, Assi S, Ouedraogo H, Rogier C (2009). Social and environmental malaria risk factors in urban areas of Ouagadougou, Burkina Faso. Malaria J.

[32] Monroe A, Moore S, Okumu F, Kiware S, Lobo NF, Koenker H (2020). Correction to: methods and indicators for measuring patterns of human exposure to malaria vectors. Malaria J.

[33] Seyoum A, Sikaala CH, Chanda J, Chinula D, Ntamatungiro AJ, Hawela M (2012). Human exposure to anopheline mosquitoes occurs primarily indoors, even for users of insecticide-treated nets in Luangwa Valley, South-east Zambia. Parasit Vectors.

[34] Soma DD, Zogo B, Taconet P, Somé A, Coulibaly S, Baba-Moussa L (2021). Quantifying and characterizing hourly human exposure to malaria vectors bites to address residual malaria transmission during dry and rainy seasons in rural Southwest Burkina Faso. BMC Public Health.

[35] Dear NF, Kadangwe C, Mzilahowa T, Bauleni A, Mathanga DP, Duster C (2018). Household-level and surrounding peri-domestic environmental characteristics associated with malaria vectors *Anopheles arabiensis* and *Anopheles funestus* along an urban-rural continuum in Blantyre, Malawi. Malaria J.

[36] MSAS (2022). Enquete sur les indicateurs du paludisme au Senegal (EIPS) 2020–2021.

[37] Lindsay SW, Thomas MB, Kleinschmidt I (2021). Threats to the effectiveness of insecticide-treated bednets for malaria control: thinking beyond insecticide resistance. Lancet Glob Health.

[38] Maxwell CA, Msuya E, Sudi M, Njunwa KJ, Carneiro IA, Curtis CF (2002). Effect of community-wide use of insecticide-treated nets for 3–4 years on malarial morbidity in Tanzania. Trop Med Int Health.

